# Endovascular Management With a Fenestration Technique of an Iatrogenic Thoracic Chronic Type B Aortic Dissection

**DOI:** 10.7759/cureus.117385

**Published:** 2026-10-03

**Authors:** Harold Mesquita da Cunha, Simon Landsweerdt, Julien Possoz, Charles Swaelens

**Affiliations:** 1 Vascular Surgery, Grand Charleroi Hospital, Charleroi, BEL

**Keywords:** aortic aneurysm repair, conservative vs. surgical management, endoleak management, endovascular aneurysm repair, type b aortic dissection

## Abstract

We report the case of an 86-year-old woman who developed an intraoperative type B aortic dissection during ascending aortic replacement for an ascending aortic aneurysm. Postoperative imaging demonstrated an uncomplicated dissection extending to the infrarenal aorta. At 10 weeks, follow-up imaging revealed rapid aneurysmal expansion of the false lumen. Thoracic endovascular aortic repair was performed with adjunctive cerebrospinal fluid drainage, rapid pacing, and image fusion guidance. An intimal fenestration technique was used to optimize the distal sealing zone, extending the disruption from a pre-existing re-entry tear. A type Ib endoleak necessitated additional stent-graft deployment, which in turn caused occlusion of the left renal and superior mesenteric arteries. The STABILISE technique (stent-assisted balloon-induced intimal disruption and relamination in aortic dissection repair) with a PETTICOAT (Provisional Extension To Induce Complete Attachment) bare stent restored renal perfusion, and targeted stenting re-established mesenteric flow. A postprocedural pseudoaneurysm was managed conservatively and resolved with spontaneous thrombosis within 48 hours. The patient was discharged on postoperative day seven, asymptomatic, with complete exclusion of the dissection, patent visceral vessels, and no endoleak confirmed at six-week follow-up. This case illustrates that combining distal flap fenestration with STABILISE relamination and a comprehensive endovascular toolkit can successfully manage complex chronic type B aortic dissections, even in elderly, high-risk patients-provided that adequate perioperative safety measures and a broad endovascular armamentarium are available to address unanticipated complications.

## Introduction

Iatrogenic type B aortic dissection (ITBAD) is a rare complication of cardiac or aortic surgery, with an incidence ranging between 0.5 and two percent [1]. Despite often being asymptomatic, persistent perfusion of the false lumen (the abnormal blood-filled channel created within the aortic wall when blood splits the media layers) is associated with aneurysmal degeneration, a progressive dilation of the aortic wall that can develop in a chronically patent false lumen. This raises the risk of aortic rupture and poor long-term outcomes [2]. Persistent flow may be sustained through the primary defect in the layers of the aortic wall, but also by a secondary intimal re-entry tear, located distally, through which blood flows back into the true lumen.

Thoracic endovascular aortic repair (TEVAR), a minimally invasive procedure in which a covered stent graft is delivered via femoral or iliac access to seal the entry tear and exclude the diseased segment of the descending thoracic aorta, has become the first-line treatment for acute thoracic aortic dissection in complicated (ruptured or pending rupture, with end-organ malperfusion, with pain or hypertension refractory to best medical treatment) or high-risk presentations. In chronic dissection, TEVAR is indicated to promote false lumen thrombosis when aneurysmal degeneration is present, with rapid growth of aortic diameter, commonly ≥ 5 mm per six months or ≥ 10 mm per year, with persistent or recurrent symptoms, or with branch vessel malperfusion [3,4]. 

We present a case of ITBAD which, 10 weeks after initial surgical repair, led to symptomatic persistent false lumen expansion through a distal re-entry tear. During the surgical procedure, two major complications occurred: an endoleak beneath one stent-graft and the loss of perfusion of two visceral arteries. This case and the way these complications were addressed highlight the importance of perioperative safety measures being taken and of having a large panel of endovascular tools permitting diagnosis and treatment of arising adverse events.

This article was previously presented as a poster at the 2026 Royal Belgian Society for Surgery (RBSS) Belgian Surgical Week on April 23, 2026.

## Case presentation

An 86-year-old woman with a history of arterial hypertension, hypercholesterolemia, and a known ascending aortic aneurysm underwent elective ascending aortic replacement. During initiation of cardiopulmonary bypass, an intraoperative type B aortic dissection occurred, requiring concomitant hemi-arch replacement.

Routine postoperative computed tomography angiography (CTA) demonstrated an aortic dissection originating at the distal aortic arch and extending to the infrarenal abdominal aorta. All visceral and both iliac arteries arose from the true lumen. There was no visceral or peripheral malperfusion. The maximum total diameter of the aorta at the time was 36 mm. There was no other major incident to be noted during the immediate postoperative period.

At the 10-week follow-up, repeat CTA confirmed persistence of the dissection, extending from the posterior aspect of the aortic arch distal to the left subclavian artery to a re-entry tear located just distal to the left renal artery. The false lumen diameter measured 35 mm, significantly exceeding the true lumen diameter (11 mm), with early aneurysmal degeneration of the dissected aorta. The aorta thus went from a maximal diameter of 36 mm to one of 47 mm. The patient reported persistent dorsal pain at that time (Figure 1).

**Figure 1:**
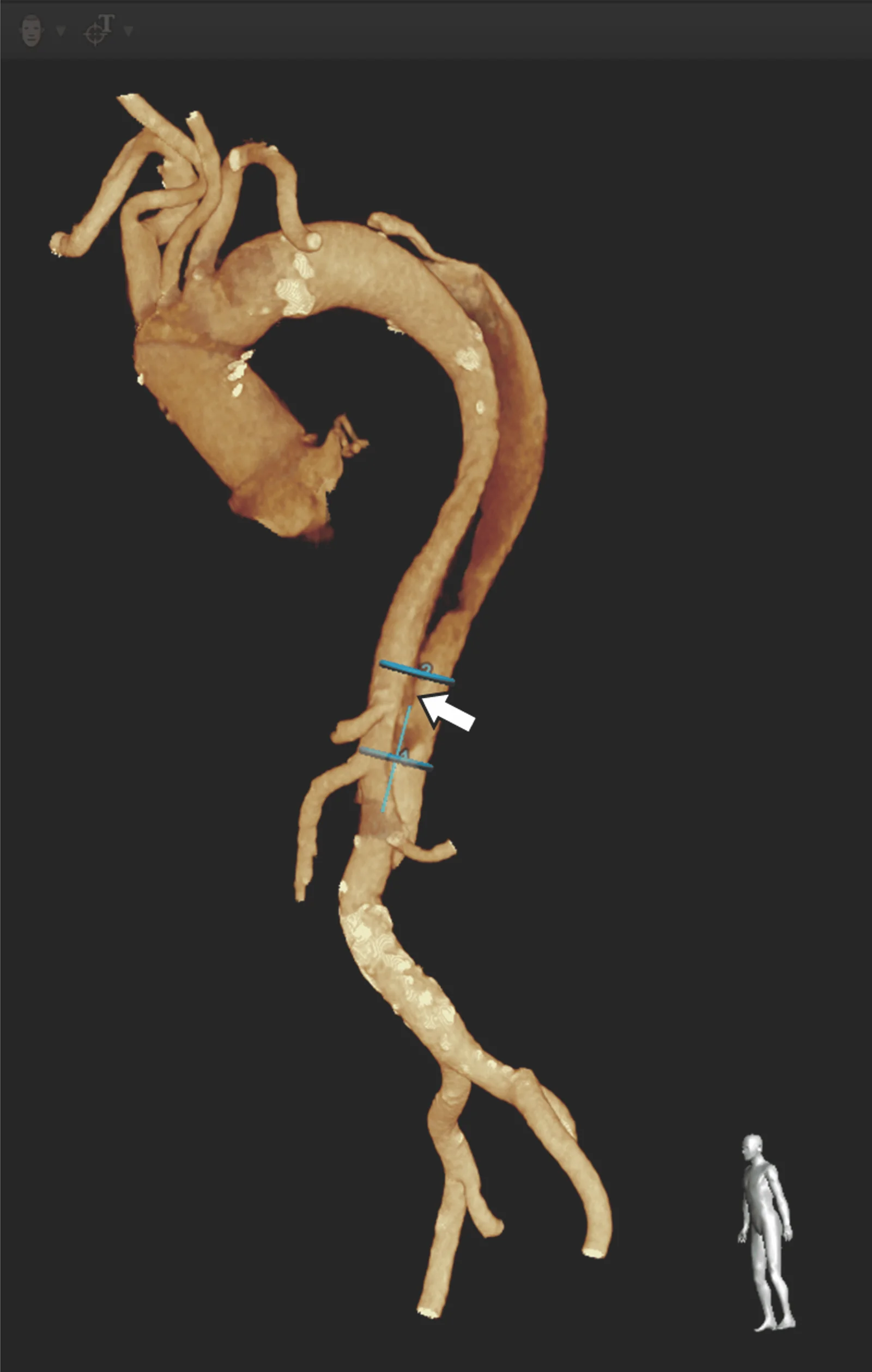
3D reconstruction of preoperative CT angiography showing the aortic dissection. The re-entry tear is located at the coeliac trunk level (arrow).

In view of the rapid aneurysmal expansion of the post-dissection aorta and ongoing symptoms despite optimal medical therapy, TEVAR was recommended. After initially declining treatment, the patient ultimately consented to the procedure, which was performed 119 days after the intraoperative onset of the dissection.

The patient underwent TEVAR with the aim of excluding the false lumen. The procedure was carefully planned to maximize procedural safety and accuracy. A preoperative cerebrospinal fluid drainage catheter was inserted to reduce the risk of postoperative spinal cord ischemia. A rapid pacing wire was also positioned to allow precise deployment of the endograft at the level of the left subclavian artery, and image fusion guidance was used to facilitate intraoperative navigation. This means that each anatomical landmark of interest for the procedure had been highlighted by a marker visible on the live cone-beam CT (CBCT): subclavian artery and proximal sealing zone; celiac trunk, re-entry tear, and expected distal sealing zone; renal arteries and the superior mesenteric artery. 

A first covered stent graft (Cook ZTA P 40-117, William Cook Europe APS, Bjaeverskov, Denmark) was deployed flush with the left subclavian artery after fusion mask alignment and under rapid pacing.

To optimize the distal sealing zone and fully exclude the thoracic aortic aneurysm, fenestration of the dissection membrane was then performed. The false lumen was catheterized from the right femoral artery, using a VanSchie 2 (Cook Incorporated, Bloomington, IN, USA) catheter advanced over a Radifocus Guide Wire (Terumo Corporation, Tokyo, Japan). The guidewire was advanced into the false lumen, allowing placement of a steerable sheath (Medtronic, Minneapolis, MN, USA) within the false lumen.

The intimal membrane was subsequently perforated using an Astato (Asahi Intecc Co., Aichi, Japan) guidewire supported by a Sergeant catheter (Life Vascular Devices Biotech S.L., Barcelona, Spain), without the use of electrocautery. The guidewire was then snared in the true lumen using a lasso device, creating a through-and-through cheese-wire configuration. Fenestration was completed by applying simultaneous traction on both ends of the guidewire under fluoroscopic guidance, extending the disruption from the perforation site to the pre-existing re-entry tear opposite the celiac trunk. Control angiography confirmed the absence of visceral malperfusion (Figure 2).

**Figure 2:**
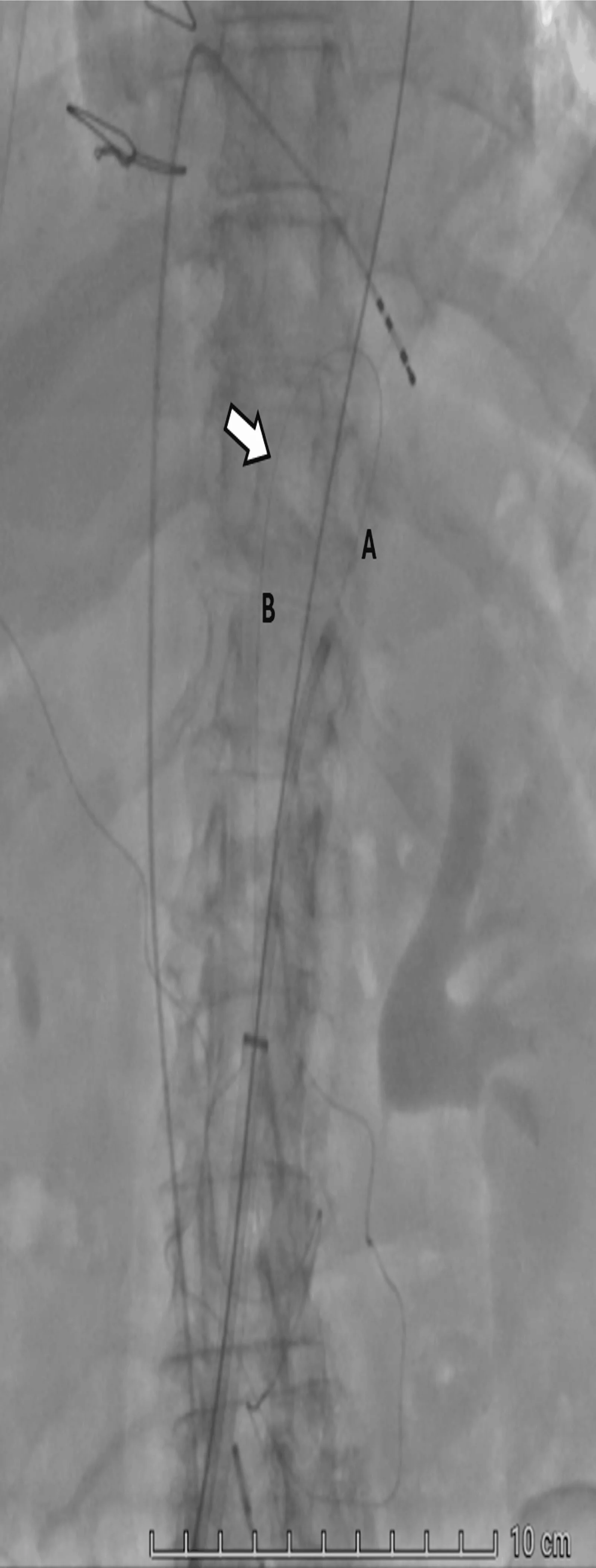
Per-operative angiography. The wire is in "cheese-wire" configuration (arrow), passing from the false lumen (A) to the true lumen (B) just before the fenestration.

A second stent graft (Cook ZTA P 40-32-165, William Cook Europe APS, Bjaeverskov, Denmark) was then deployed within the newly optimized distal sealing zone. Completion angiography revealed a type Ib endoleak, prompting deployment of a third stent graft (Cook ZTA P 34-161, William Cook Europe APS, Bjaeverskov, Denmark). Subsequent angiography demonstrated resolution of the endoleak but also revealed loss of perfusion of the left renal artery and the superior mesenteric artery (SMA).

The STABILISE technique (stent-assisted balloon-induced intimal disruption and relamination in aortic dissection repair) was then performed using a PETTICOAT (provisional extension to induce complete attachment) (Cook ZDES 38-180, William Cook Europe APS, Bjaeverskov, Denmark) bare stent, dilated with a non-compliant Atlas GOLD 26-40 balloon (Bard Peripheral Vascular, Tempe, AZ, USA). This maneuver restored perfusion to the left renal artery, but not to the SMA (Figure 3).

**Figure 3:**
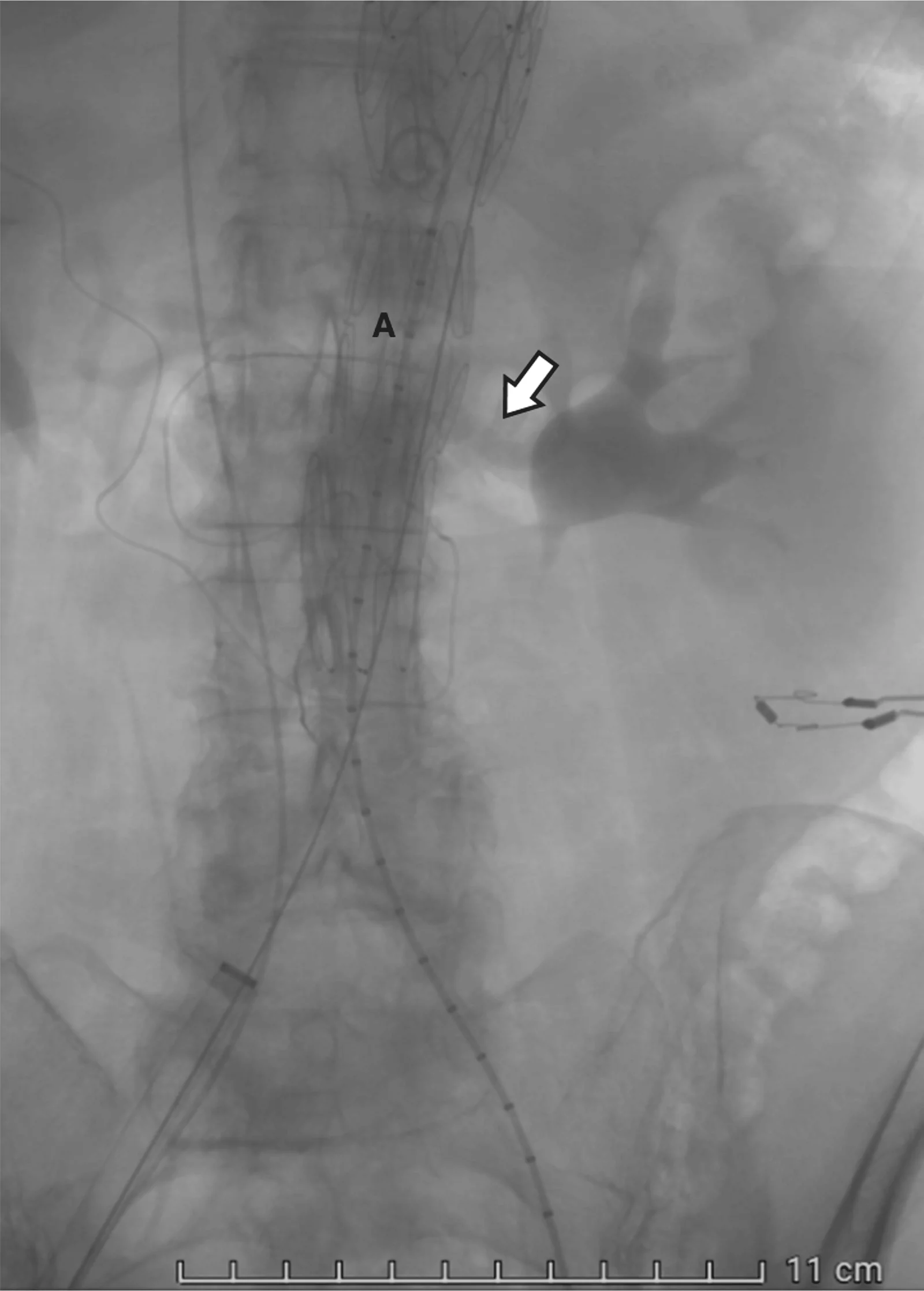
Per-operative angiography. Restored left renal artery (arrow) after the use of the STABILISE (stent-assisted balloon-induced intimal disruption and relamination in aortic dissection repair) technique using a bare stent (A) and a non-compliant balloon.

The SMA was subsequently catheterized using fusion guidance and revascularized with a Restorer 7 × 38 mm stent (Life Vascular Devices Biotech, Barcelona, Spain), reinforced with an Absolute Pro 8 × 40 mm stent (Abbot Medical, Santa Clara, CA, USA) (Figure 4).

**Figure 4:**
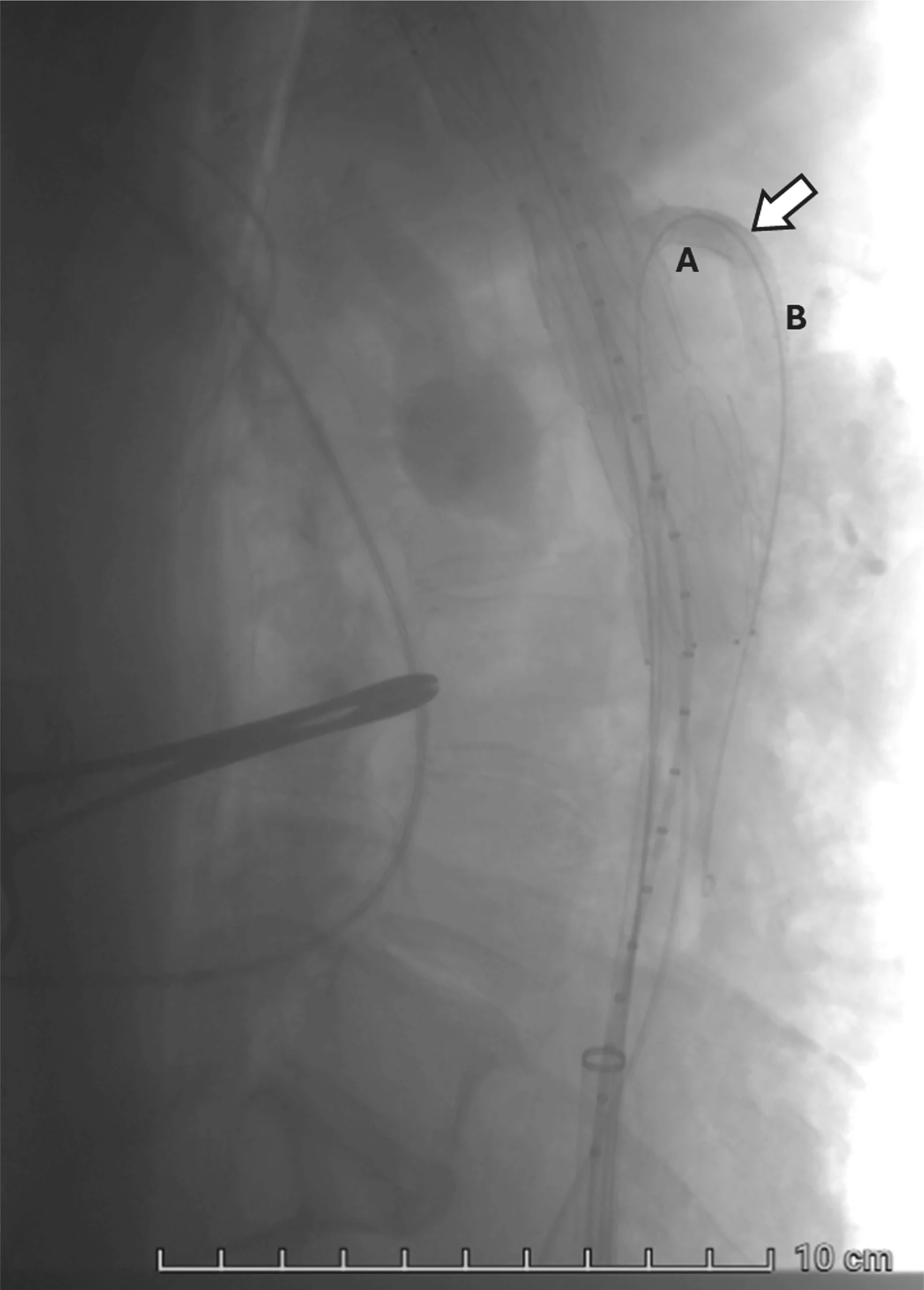
Per-operative angiography. Restored superior mesenteric artery (SMA) (arrow) following stenting with a balloon-expandable stent (A) reinforced by a self-expanding stent (B).

Final angiography demonstrated adequate perfusion of all four visceral arteries. Femoral access sites were closed using ProStyle closure devices (Abbot Medical, Santa Clara, CA, USA), which had been pre-positioned at the beginning of the procedure.

In the immediate postoperative period, the patient developed acute abdominal pain, prompting urgent CTA. Imaging revealed limited contrast extravasation near the origin of the renal arteries, consistent with a postprocedural pseudoaneurysm.

Given the technical complexity of reintervention, the patient’s advanced age and frailty, and her hemodynamic stability, a conservative management strategy was adopted. 

A follow-up CT scan performed three hours later showed no progression of the extravasation, accompanied by clinical improvement of the abdominal symptoms. A subsequent CT scan at 72 hours demonstrated complete thrombosis of the pseudoaneurysm, without further contrast extravasation. Imaging also confirmed complete exclusion of the dissection and absence of endoleak (Figure 5).

**Figure 5:**
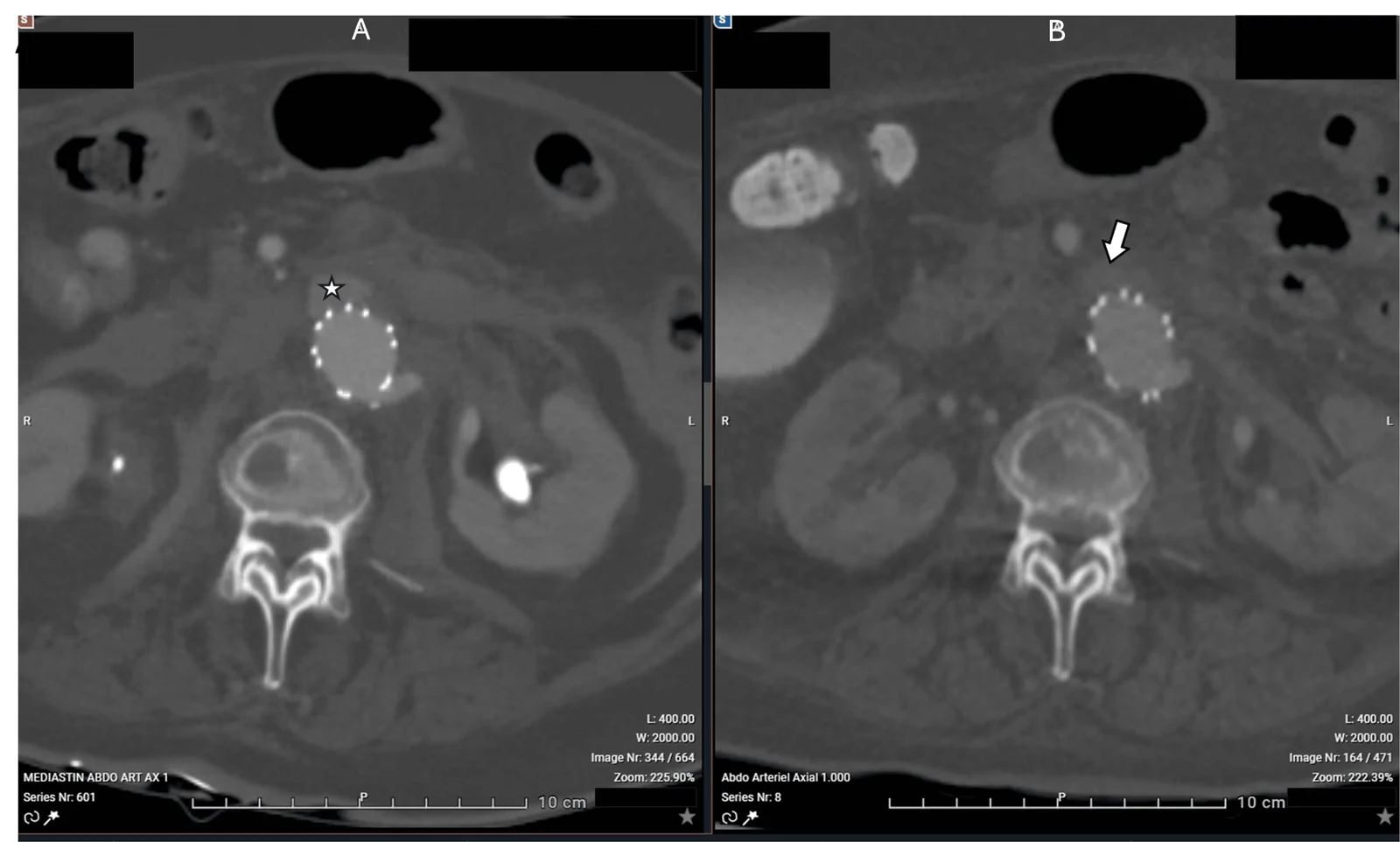
Axial contrast-enhanced CT scans (three days apart). (A) Active contrast extravasation (star) consistent with a leak at the level of the right renal artery. (B) Complete resolution of the extravasation (arrow), confirming the absence of persistent leakage.

The patient was discharged home on postoperative day seven, asymptomatic. At the six-week clinical and radiological follow-up, the patient was asymptomatic, and CTA showed complete aortic remodeling, patency of all visceral arteries, and no evidence of endoleak (Figure 6).

**Figure 6:**
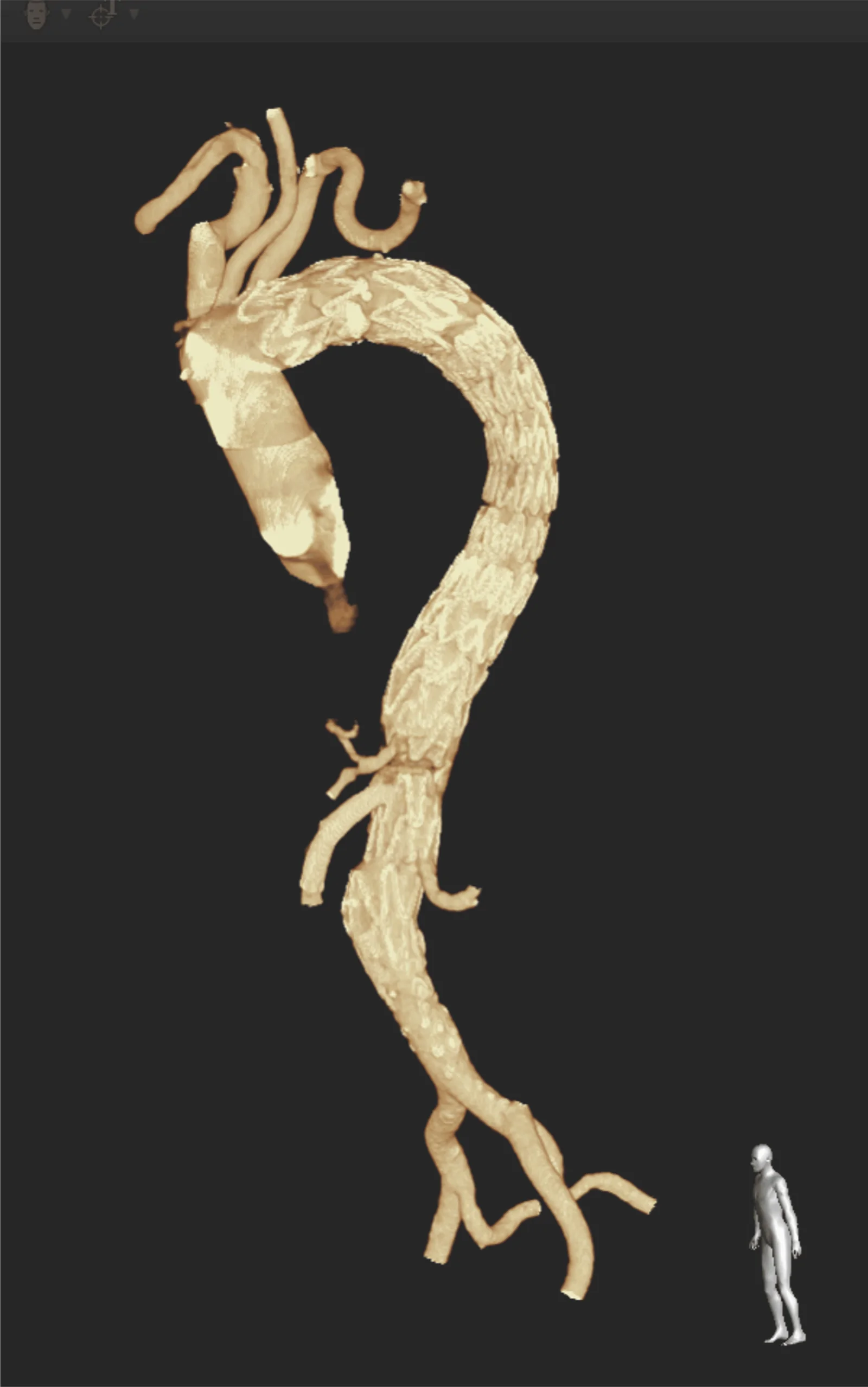
3D reconstruction of six-week postoperative CT angiography. Complete exclusion of the aortic dissection. All visceral arteries are patent. No evidence of endoleak.

## Discussion

Type B aortic dissections originate distal to the left subclavian artery and are classified by timing (acute, subacute, and chronic) and by complication status [5]. Complicated dissections involve rupture, refractory pain/hypertension, rapid aneurysmal growth, malperfusion, or spinal cord ischemia [5,6]. Initial treatment is medical (pain and blood pressure control), but complicated cases - as in this patient, with rapid aneurysm growth and persistent pain - warrant intervention [7]. TEVAR has become the preferred approach for early chronic dissections, before flap thickening compromises graft apposition; here, the patient's age, favorable anatomy, and lack of connective tissue disease supported this choice [8,9].

The originality of this case lies primarily in the use of intimal flap fenestration to optimize the distal sealing zone of the stent graft, in order to exclude aortic aneurysm [10,11]. This strategy aims to increase the length of apposition within the true lumen, promote aortic remodeling, and reduce the risk of distal endoleak [12]. However, it carries a potential risk of creating a free-floating intimal flap, as illustrated here by the secondary occlusion of the ostia of the SMA and left renal artery [13]. The STABILISE technique, combined with placement of a PETTICOAT stent, allowed the flap to be re-apposed against the aortic wall and restored renal perfusion [14,15], while targeted stenting of the SMA corrected the mesenteric malperfusion.

This case also underscores the importance of preoperative planning and advanced navigation tools (image fusion, precise planning of access routes and devices) for managing complex anatomy and unanticipated intraoperative complications [16,17]. Indeed, it is through precise analysis of the preoperative CT angiograms, and the fusion of those images with the peroperative CBCT live rendering, that the SMA was cannulated although there was no stump visible on the angiograms during the operation.

The use of prophylactic spinal drainage illustrates the need to anticipate neurologic risk during extensive thoracoabdominal aortic repairs. Seeing as the dissection extended from the subclavian artery to a level just upstream of the celiac trunk, the aortic coverage had to be extensive in order to fully exclude the false lumen. Given that length, the number of intercostal arteries to be sacrificed, the coverage of the aortic segments between the eighth and twelfth vertebrae, and the advanced age of the patient at the time of the surgery, prophylactic cerebrospinal fluid drainage was performed [18,19].

Finally, the conservative management of a post-procedural pseudoaneurysm in a frail but hemodynamically stable patient highlights the value of an individualized assessment of the risk-benefit ratio before pursuing invasive reintervention. The therapeutic options were limited in this elderly patient, as well as in this setting. Other than the option of open surgical repair, endovascular possibilities were an endograft with in-situ laser fenestration, or the use of the chimney technique. 

There was, however, a high risk that endovascular coverage, with the need to balloon the aortic wall, might cause further rupture. The open surgical approach was considered too dangerous for the patient. The watchful waiting approach was deemed successful at confirmation of the spontaneous thrombosis of the pseudoaneurysm 72 hours later, thus avoiding an additional and potentially major procedure while still ensuring effective exclusion of the dissection.

This case suggests that fenestration of the distal sealing zone, combined with relamination techniques such as STABILISE and a comprehensive endovascular armamentarium, may offer an effective solution for complex chronic type B aortic dissections [20].

## Conclusions

This single clinical case underscores how iatrogenic type B aortic dissection, while uncommon, can evolve into a complex aneurysmal process requiring advanced endovascular strategies in addition to standard TEVAR. The combination of distal intimal flap fenestration to optimize the sealing zone, together with STABILISE-based relamination and targeted visceral stenting, allowed successful exclusion of the dissection despite the challenges posed by visceral malperfusion during the procedure.

This experience, although not sufficient to draw general conclusions for every similar clinical presentation, highlights three key principles for managing similarly complex cases: First, meticulous perioperative planning, including spinal cord protection, rapid pacing, and image fusion guidance, is essential and may be particularly useful when anatomy is unfavorable or complications are anticipated. Second, a comprehensive endovascular armamentarium must be available to promptly address adverse events such as endoleak or malperfusion arising intraoperatively. Third, conservative management of postprocedural complications, such as a stable pseudoaneurysm, may be appropriate in frail patients when invasive reintervention carries disproportionate risk.
